# Rare Adult-onset Citrullinemia Type 1 in the Postpartum Period: A Case Report

**DOI:** 10.5811/cpcem.2022.10.57277

**Published:** 2023-01-24

**Authors:** Michael Borsuk, Mathew Saab, Michael Tobin

**Affiliations:** * Madigan Army Medical Center, Tacoma, Washington; † Madigan Army Medical Center, Department of Emergency Medicine, Tacoma, Washington

**Keywords:** case report, citrullinemia type 1, urea cycle disorder, hyperammonemia

## Abstract

**Introduction:**

Citrullinemia type 1 (CTLN1) is a urea cycle disorder caused by defective argininosuccinate synthetase leading to impaired ammonia elimination. Urea cycle disorders are typically diagnosed on neonatal screening but rarely can lie dormant until a metabolic stressor causes initial onset of symptoms in adulthood.

**Case Report:**

A 23-year-old female presented four days postpartum to the emergency department (ED) obtunded and declined to the point of requiring intubation. Labs revealed hyperammonemia, and she was subsequently found to have CTLN1.

**Conclusion:**

Urea cycle disorders presenting in adulthood are a rare etiology for the common ED complaint of altered mental status. The low incidence makes these treatable disorders easy to overlook leading to potentially significant morbidity and mortality. Therefore, it is important to recognize the risk factors that can trigger an acute metabolic derangement. This case highlights common risk factors for metabolic stress, possible presenting symptoms, and the positive outcome achievable when recognized and treated in a timely fashion.

## INTRODUCTION

Citrullinemia type 1 (CTLN1) is one of six principal urea cycle disorders (UCD) most often diagnosed during infancy. Despite all 50 states in the United States currently screening for CTLN1, many silent or mild cases are likely not detected with current methods.^1^ Rarely, UCDs present in adulthood following a stressor or metabolic change causing the rate of ammonia production to exceed the rate of urea metabolism. When the defective enzyme cannot adequately compensate, the imbalance may manifest with the clinical features lethargy, slurred speech, cerebral edema, and asterixis.^2^ With altered mental status being a frequent emergency department (ED) complaint and often not allowing for a complete history, UCDs pose a difficult and rare diagnostic challenge to the emergency physician.

Prompt recognition and dietary changes portend a good prognosis, while untreated accumulation of ammonia can result in death or permanent disability.^2^ Currently, the diagnosis can be made with urine and plasma amino acid levels, and confirmed with either tissue enzyme activity testing or argininosuccinate synthetase 1 gene mutation analysis.^3^ None of these results are immediately available to the emergency physician making a definitive diagnosis impossible. Therefore, prompt treatment requires recognizing metabolic risk factors and obtaining proper labs. Often, an elevated ammonia level will point to the diagnosis and allow for empiric hyperammonemia treatment. We present a case of a postpartum mother in the ED with altered mental status subsequently discovered to have CTLN1.

## CASE REPORT

A 23-year-old female presented via emergency medical services (EMS) for slurred speech, irregular behavior, and vision changes. Initial vitals were blood pressure 122/78 millimeters of mercury, heart rate of 58 beats per minute (min), respiratory rate of 14 breaths per min, oxygen saturation of 98% on room air, and rectal temperature of 35.8 C. She was opening her eyes to pain, yelling incomprehensibly, and experiencing localized pain for a Glasgow Coma Scale of 9. Per EMS report, she had been in her usual state of health 10 hours previously after driving herself home from visiting her newborn in the neonatal intensive care unit (NICU). She was found at home by her spouse speaking unintelligibly and appeared lethargic.

Initial EMS scene assessment noted dilated pupils bilaterally, confused speech, and somnolence with a complaint of scotomas. History obtained from family revealed a remote history of seizures currently not requiring an antiepileptic. She had also delivered four days prior at 34 weeks and four days gestation due to preterm premature rupture of membranes (PPROM). Family history was noncontributory. Remarkable findings on ED exam include clammy, pale skin, 6-millimeter bilateral reactive pupils, and appropriate reflexes, but she was uncooperative with full neurological exam. Electrocardiogram displayed sinus bradycardia in a nonischemic pattern.

Laboratory studies revealed aspartate aminotransferase (AST) 86 units per liter (U/L) (reference range: 8–36 U/L); alanine transaminase (ALT) 97 U/L (4–36 U/L); alkaline phosphatase 209 U/L (20–130 U/L); albumin 2.6 grams per deciliter (g/dL) (34–54 g/dL); total protein 5.4 g/dL (6.0–8.3 g/dL); blood urea nitrogen 4.0 milligrams (mg)/dL (6–20 mg/dL); and bilirubin 0.30 mg/dL (0.1–1.2 mg/dL). Serum lactic acid was 2.3 millimoles per liter (mmol/L) (0.5–2.2 mmol/L); international normalized ratio (INR) was 2.2; haptoglobin 98 mg/dL (30–200 mg/dL); and ammonia level was 158 micromoles (μmol)/L (11–32 μmol/L). A urine drug screen was negative. Serum samples contained undetectable ethanol, salicylate, and acetaminophen levels. Imaging ordered included a non-contrast computed topography (CT) head and a contrast CT of the abdomen and pelvis that displayed no acute intracranial or intra-abdominal processes. Abdominal ultrasound revealed nonspecific fatty changes in the liver. Due to rapidly declining mentation with inability to protect her airway she was intubated and admitted to the intensive care unit (ICU) on empiric antibiotics.

In the ICU, lactulose therapy was started for hepatic encephalopathy secondary to suspected fatty liver of pregnancy. She was continued on broad spectrum antibiotics and antivirals for encephalitis coverage until completion of an unremarkable brain magnetic resonance imaging, extubation, and mental status improvement on ICU day two. Serum ammonia continued to rise to 400 μmol/L despite lactulose therapy and steadily improving neurological status over the following days. Gastroenterology consultation suggested the possibility of a UCD and recommended serum and urine amino acid testing.

On admission day seven, urine amino acid results showed elevated citrulline at 17,677 μmol/g creatine (μmol/g cr) (Ref: 1.0–27.4 umol/g cr). On hospital day eight, genetic testing revealed two separate mutations of the *ASS1* gene encoding argininosuccinate synthetase, confirming the diagnosis of CTLN1. Management was switched from lactulose therapy to sodium phenylbutyrate and arginine. Despite downtrending ammonia levels, liver function tests (LFT) continued to climb to a peak of ALT 1,868 U/L and AST of 1745 U/L on day nine, prompting transfer to a transplant center. At the transplant center, the patient remained asymptomatic and LFTs downtrended until she was eventually discharged on sodium phenylacetate-sodium benzoate, a strict low-protein diet, and recommendation to avoid future pregnancy.

*CPC-EM Capsule*

What do we already know about this clinical entity?

*Citrullinemia is an autosomal recessive disorder that is caused by impaired argininosuccinate synthetase. The majority of cases are diagnosed in infancy.*

What makes this presentation of disease reportable?

*It is not commonly known that one may live completely asymptomatically with the milder forms of this condition until a metabolic stressor.*

What is the major learning point?

*Recognize metabolic stressors such as pregnancy as compared to the presentation with other liver diseases that present in the peripartum period.*

How might this improve emergency medicine practice?

*This case shows the importance of keeping a broad differential when the presentation does not fit a more commonly seen pattern.*

## DISCUSSION

Urea cycle disorders are better known to pediatric physicians as they most commonly present in the neonatal period and are thought to occur in roughly 1/8,000 births.^4^ With unreliable screening mechanisms for mild or silent forms, many remain undetected until severe presentation with severe multiorgan dysfunction in adulthood following metabolic stress.^1^ While this case included prompt supportive care and admission to the ICU, any prolonged boarding time, healthcare access issues, or failure to consider this rare diagnosis may have progressed to fulminant liver failure.

Citrullinemia type 1 and other hereditary enzyme deficiencies can be exacerbated in the setting of an acute stressor that causes increased metabolism. Aside from the more likely considered diagnoses, the potential for an underlying predisposition to metabolic derangement in adulthood should be considered by the emergency physician. This may allow for better direction of treatment by the admitting service or consultant. These treatments are often simple to implement. For example, in milder CTLN1 only dietary changes are necessary. Understanding UCDs and treatment requires understanding the deficient enzyme’s role.

The urea cycle eliminates ammonia from protein breakdown by converting it to urea for excretion in the urine. Catabolic metabolic states increase the production of ammonia from protein breakdown that can potentially overload the less abundant or defective enzyme in pathologic states. Conditions that cause increased protein breakdown include high protein diet, prolonged starvation, infection, drugs, and physical exertion. This patient had significant metabolic changes prior to presentation given her recent PPROM delivery and the associated highly catabolic state. Likewise, she potentially underwent decreased oral intake in the days prior to her ED presentation, given her frequent visits to the NICU, and later during her subsequent ICU admission. This case exemplifies why it may be efficacious to acquire a serum ammonia level as part of the workup in patients exhibiting altered mental status with elevated LFTs and no known history of liver disease.

In this case, metabolic disorders were considered when the common etiologies were excluded. This included ruling out hypoglycemia, normal imaging ruling out any acute intracranial or intra-abdominal processes, low suspicion for ingestion given no toxidrome and negative toxicology labs, and no significant electrolyte abnormalities. The lab abnormalities did not fit a primary epileptic or psychological etiology. The diagnosis came down to differentiating a metabolic disorder from other liver diseases in pregnancy such as hemolysis, elevated liver enzymes, low platelets (HELLP) syndrome, eclampsia, and acute fatty liver of pregnancy (AFLP).

Unfortunately, metabolic disorders presenting as liver failure share many features with other diseases in pregnancy. The main three to consider are eclampsia, HELLP syndrome, and AFLP. There are many overlapping features because all can present elevated LFTs and liver synthesis abnormalities seen on labs. One of the first ways to differentiate is blood pressure. Hypertension is almost always present in eclampsia and usually present in HELLP and AFLP. Next is to look for hemolysis. This case showed elevated liver enzymes, elevated ammonia, elevated INR, and elevated lactate dehydrogenase that presented a potential hemolytic picture also seen in HELLP and AFLP. Unlike in patients with HELLP and AFLP, our patient was normotensive with normal platelets and haptoglobin. In this case a peripheral smear would have been useful to look for schistocytes. It is also important to note that fatty changes in the liver as seen in this case are not specific and not diagnostic for AFLP.

It may be unreasonable to differentiate these different conditions with certainty in the ED; however, the emergency physician should consider metabolic disorders because there are interventions that if initiated early can alter the clinical course. In this case, intubation and sedation likely decreased metabolic activity and ammonia load allowing the urea cycle to achieve homeostasis. The reasonable clinical decision to keep her nil per os until resuming a regular diet prevented further protein introduction and subsequent ammonia production. Conversely, the absence of carbohydrates ensured continued catabolism for gluconeogenesis. The net effect of these counterbalancing considerations cannot be measured except by clinical improvement.

If immediate transfer or admission is not possible, management must start in the ED. A rapidly declining patient might prompt consideration of dialysis for hyperammonemia. However, emergent dialysis also removes glucose that can worsen the catabolic state if not repleted. Frequent monitoring of ammonia levels is recommended since reversal of the metabolic state often takes multiple days. The recommended treatment for undifferentiated UCDs is sodium phenylacetate-sodium benzoate and arginine, which works by binding ammonia for removal and shunting ammonia down an alternate pathway from the urea cycle.^3^ If sodium phenylacetate-sodium benzoate is not available, transfer may be considered while awaiting results of the urine/serum amino acid levels or genetic testing.

## CONCLUSION

Citrullinemia type 1 and other underlying metabolic defects must be considered by the emergency physician caring for a patient such as in this case. This case serves as an example to maintain a wide differential and obtain appropriate testing when the history, exam, and initial labs demonstrate peculiarities that may demonstrate a metabolic abnormality. Were her presentation milder, not warranting intubation and ICU admission, her course may have continued with progressive undiagnosed hepatic dysfunction. Luckily for this patient, she recovered thanks to prompt discovery in her obtunded state by her husband, and successful diagnosis by our critical care and gastroenterology colleagues. In the future, improved genetic testing and genome mapping may make awareness of these indolent metabolic decompensations more apparent and less frequent. In the meantime, as technology races toward these solutions, astute emergency physicians can provide the initial stabilization and work-up as they have for decades with similarly undifferentiated patients.
